# Hypoxia-activated selectivity-improved anti-PKM2 antibody combined with prodrug TH-302 for potentiated targeting therapy in hepatocellular carcinoma

**DOI:** 10.7150/ijbs.92211

**Published:** 2024-02-11

**Authors:** Bo Wang, Fang-Zheng Qi, Ping Chen, Luo-Meng Qian, Hui-Shan Su, Yang Wang, Chen-Hui Wang, Ya-Xin Hou, Qing Zhang, Ding Li, Zhe-Sheng Chen, Si-He Zhang

**Affiliations:** 1 Department of Cell Biology, School of Medicine, Nankai University, Tianjin, 300071, China.; 2 National Clinical Research Center for Cancer, Key Laboratory of Cancer Prevention and Therapy, Tianjin Medical University Cancer Institute and Hospital, Tianjin, 300060, China.; 3 Department of Pharmaceutical Sciences, College of Pharmacy and Health Sciences, St. John's University, Queens, New York, NY, 11439, USA.

**Keywords:** Hypoxia-activated antibody, On-target off-tumor toxicity, TH-302, PKM2, Hepatocellular carcinoma

## Abstract

**Background:** Hypoxia induces hepatocellular carcinoma (HCC) malignancies; yet it also offers treatment opportunities, exemplified by developing hypoxia-activated prodrugs (HAPs). Although HAP TH-302 combined with therapeutic antibody (Ab) has synergistic effects, the clinical benefits are limited by the on-target off-tumor toxicity of Ab. Here, we sought to develop a hypoxia-activated anti-M2 splice isoform of pyruvate kinase (PKM2) Ab combined with TH-302 for potentiated targeting therapy.

**Methods:** Codon-optimized and hypoxia-activation strategies were used to develop H103 Ab-azo-PEG5k (HAP103) Ab. Hypoxia-activated HAP103 Ab was characterized, and hypoxia-dependent antitumor and immune activities were evaluated. Selective imaging and targeting therapy with HAP103 Ab were assessed in HCC-xenografted mouse models. Targeting selectivity, systemic toxicity, and synergistic therapeutic efficacy of HAP103 Ab with TH-302 were evaluated.

**Results:** Human full-length H103 Ab was produced in a large-scale bioreactor. Azobenzene (azo)-linked PEG5k conjugation endowed HAP103 Ab with hypoxia-activated targeting features. Conditional HAP103 Ab effectively inhibited HCC cell growth, enhanced apoptosis, and induced antibody-dependent cellular cytotoxicity (ADCC) and complement-dependent cytotoxicity (CDC) functions. Analysis of HCC-xenografted mouse models showed that HAP103 Ab selectively targeted hypoxic HCC tissues and induced potent tumor-inhibitory activity either alone or in combination with TH-302. Besides the synergistic effects, HAP103 Ab had negligible side effects when compared to parent H103 Ab.

**Conclusion:** The hypoxia-activated anti-PKM2 Ab safely confers a strong inhibitory effect on HCC with improved selectivity. This provides a promising strategy to overcome the on-target off-tumor toxicity of Ab therapeutics; and highlights an advanced approach to precisely kill HCC in combination with HAP TH-302.

## Introduction

The mortality rate of hepatocellular carcinoma (HCC) continues to increase worldwide 1, 2. For HCC patients at an early-stage, resection, liver transplantation and local ablation are appropriate; however, early detection is challenging. Chemoembolization is suitable for treating HCC at an intermediate-stage, but it has numerous side effects. Systemic targeted therapies, including kinase inhibitors, anti-VEGF antibodies (Abs) and immune checkpoint inhibitors (ICIs), are available for advanced-stage HCC. However, the clinical outcome remains modest 1, 2. Thus, seeking new therapeutics against HCC is highly important.

Hypoxia is a unique microenvironmental feature of HCC. It promotes aggressive phenotypes that increase resistance to apoptosis and favor HCC metastasis 3-8. Hypoxia-induced HCC malignancies contribute to poor clinical prognosis. As a result, hypoxia-targeted therapies, including hypoxia-selective gene therapy and hypoxia-activated prodrug (HAP) have been developed 9. HAPs are drugs that are specifically reduced by reductases under hypoxia. Once activated, the products exhibit cytotoxicity to hypoxic cancer cells with minimal effects on normal cells. TH-302 (evofosfamide), a second-generation HAP, has high hypoxia-selectivity 10. It comprises a dual moiety of bromo-isophosphoramide (Br-IPM), a DNA cross-linking mustard prodrug, and 2-nitroimidazole, a bioreductive phosphosporamide prodrug. Both of these agents undergo reductive reactions to activate prodrugs in a hypoxic microenvironment 5, 11, 12. Although TH-302 exhibits broad-spectrum preclinical activity against cancer 13-15, its clinical trials alone and in combination with chemotherapeutics are limited by drug-related toxicity 16, 17. Synergistic therapeutics, such as TH-302 combined with the small molecule targeting drug sorafenib, have potent benefits for HCC patients. However, dose-limiting toxicities (DLTs), including fatigue, hand-foot syndrome, hypertension, nausea and vomiting, have been commonly reported 12. This finding promotes further exploitation of TH-302 combined with immunotherapy.

Accumulating studies have concentrated on the synergistic effect of TH-302 combined with Ab-based immunotherapy. Combined therapy with TH-302 and a maximal checkpoint blockade by both anti-CTLA-4 and anti-PD-1 Abs dramatically sensitizes therapy-resistant "cold" prostate cancers to immunotherapy 18. Compared with single use either alone, combined therapy with TH-302 and an anti-CTLA-4 Ab augments CTLA-4 blockade in a head and neck squamous cell carcinoma (HNSCC) model; and further improves the survival of HNSCC patients 19. Combining TH-302 with the proapoptotic receptor agonist drozitumab (anti-DR5 Ab) reduces the tumor burden in osteosarcoma 20. Combining TH-302 with bevacizumab (anti-VEGF Ab) significantly improved the progression-free survival of bevacizumab-refractory glioblastoma patients 21, 22. When TH-302 is combined with another ICI, Ipilimumab, for treating both "cold" and "warm" solid malignancies, it only achieves a partial response in patients 23. These clinical trials indicate that TH-302 can sensitize therapeutically resistant cancers to Ab-based immunotherapy. Although the clinical outcome of these explorations may be beneficial, therapy-related toxicities are inevitable. Because the TH-302 dosage used in these combined immunotherapies was already adjusted to a well-tolerated level 18-21, most drug-related adverse events observed in trials are actually derived from therapeutic Abs. This is not hard to understand, as therapeutic Abs that target an antigen (Ag) on tumor cells can also recognize the same Ag on nontumor cells, resulting in on-target off-tumor toxicity 24. Hence, how to overcome Ab-related toxicity before combination with TH-302 for synergistic therapy is urgently needed.

In this study, we utilized the hypoxic microenvironment of HCC to develop a conditional Ab based on our previously developed human anti-M2 splice isoform of pyruvate kinase (PKM2) Ab 25. This hypoxia-activated anti-PKM2 Ab was designed by covalently conjugating poly ethylene glycol (PEG) via an azobenzene (azo)-containing linker. As hypoxic cells produce nitroreductase (NTR) at a level 100-1000-fold greater than that in normoxic cells, which could reduce azo-containing drugs available for conditional release 26-28, and enable the azo-linked PEGylated anti-PKM2 Ab selectively targets hypoxic HCC cells. When this hypoxia-activated anti-PKM2 Ab was combined with hypoxia-activated TH-302, a synergistic “double kill” effect with improved targeting selectivity was highlighted.

## Materials and methods

### Cell culture, Abs, kits and chemicals

Human HCC cell lines HepG2 and Huh7 were obtained from the Type Culture Collection of the Chinese Academy of Sciences (China), and were routinely cultured under normoxic (5% CO_2_) or hypoxic (O_2_<1%) conditions (Thermo).

Human anti-PKM2 scFv Ab (H103) with internalizing property and PKM2 Ag were originally produced in our laboratory 25. Mouse anti-PKM2 mAb (sc-365684) was obtained from Santa Cruz. Rabbit anti-Hif1a Ab (340462), and anti-CD31 Ab (347526) were obtained from ZenBio. Mouse anti-β-actin Ab (KM9001) and goat anti-human IgG(H+L)-HRP (LK2005) were obtained from Sungene Biotech. Goat anti-human IgG(H+L)-RDM (109-295-003) was obtained from Jackson. Goat anti-rabbit IgG(H+L)-AF488 (A-11001) and BCA protein assay kit (23225) were obtained from Life. Human IgG (hIgG, SP001), Ficoll (P4360), RBC lysis buffer (R1010), and PMSF (329-98-6) were obtained from Solarbio. Enhanced cell counting kit-8 (CCK-8, C0038), Annexin V-PI apoptosis kit (C1062L) and LDH cytotoxicity kit (C0016) were obtained from Beyotime. Sensor CM5 chips (BR100399) were obtained from Cytiva. Cy7 dye (RH-7775) was obtained from Ruixibio. Hypoxyprobe Plus Kit (HP2-100, FITC-pimoniazole) was obtained from Hypoxyprobe. PEG5k (9004-74-4) was obtained from Fchem. 5-amino-1-pentanol (A67299) was obtained from Innochem. Na_2_S_2_O_4_ (7775-14-6) was obtained from Adamas. TH-302 (M2206) was obtained from AbMole. N-bromosuccinimide (NBS, 128-08-5), 2,4,6-trinitrobenzenesulfonic acid (TNBSA, 2508-19-2), Hoechst 33342 (B2261), and other chemicals were obtained from Sigma.

### Synthesis of amine-reactive PEG5K-azo ester

Amine-reactive PEG5K-azo ester was synthesized as indicated previously 28, 29. The procedure is briefly described in the supporting information, and the synthesis was performed by Thinkerytech Technology Co. Ltd (Tianjin, China). PEG5K-azo ester (compound #7) was synthesized by coupling PEG-azo (compound #6) with N,N'-disuccinimidyl carbonate (DSC). ^1^H NMR spectra were obtained on a Bruker Av400 instrument (Switzerland) to monitor all intermediate products.

### Plasmid construction and Ab expression

The expression and purification of the Abs were conducted by SinoBiological Co. Ltd (Beijing, China). Briefly, sequence-optimized VH and VL genes (Supplementary Table. S1) were amplified by PCR using the pCANTAB/H103-scFv plasmid as a template 25. Then, the H103-VH and H103-VL gene fragments were double-digested (Kpn I and Xba I), purified and inserted into vectors containing the constant region of human IgG1 to obtain the recombinant plasmids pcMV3-H103-H and pcMV3-H103-L, respectively. For transient expression, the two plasmids were simultaneously electroporated into HEK293 cells, and Ab-expressing clones were screened by stepwise increase in the hygromycin concentration followed by subcloning process. The Ab-secreting cells were cultured in serum-free medium and maintained in a bioreactor at a suitable stirring speed at 37 °C for six days. The culture supernatants were harvested, centrifuged, and loaded onto an affinity purification column for Ab purification at an appropriate flow rate. Finally, the purified Ab concentration was determined via SDS-PAGE.

### Ab modeling and accessible lysine prediction

H103 Ab was modeled by the canonical structure method with the PIGS program 30. Amine-reactive PEG polymers were modeled on accessible lysine residues of Ab as described previously 31. The accessible surface area (ASA) and solvent-accessible surface area (SASA) were calculated using Amber18 software. The SASA was calculated by using the surf module with the linear combination of pairwise overlaps (LCPO) method. The connolly surface area (CSA) was calculated with the molsurf module 32.

### Generation, cleavage and purification of H103-azo-PEG5K (HAP103) Ab

H103 Ab and excess PEG5K-azo ester were mixed in carbonate buffer (0.1 M, pH=9.0). After overnight-shaking (200 rpm) for reaction at 4 °C, the Ab was purified from the mixture by Amicon Ultra® centrifugal filter units (MWCO 10K). Equal volume of HAP solution (200 uM) were incubated with Na_2_S_2_O_4_ (100 μM) freshly prepared with carbonate buffer (pH=8.0) at RT for 2 h. After ultrafiltration, the Ab samples were harvested in PBS and aliquoted at -80 °C.

### SDS-PAGE, Coomassie blue staining and Western blotting

The purified HAP103 Ab was analyzed via SDS-PAGE gels with samples loaded with reducing or nonreducing buffer. After staining with Coomassie blue, the gel was washed with distaining solution overnight, and the results were recorded with a ChemiDoc XRS system.

HCC cells were lysed in RIPA buffer. The samples were quantified by a BCA kit, resolved by SDS-PAGE, transferred onto a PVDF membrane, blotted with mouse anti-PKM2 (1:1000), anti-β-actin (1:4000) and anti-Hif1a (1:500) PcAbs, and finally detected with HRP-labeled secondary Abs.

### Ab-conjugation determined by the TNBSA assay

HAP103, Na_2_S_2_O_4_-cleaved HAP103 Ab (act-HAP103) solution, or 5-amino-1-pentanol (used as a standard) was gradient-diluted in NaHCO_3_ buffer (pH=8.5), and mixed with 0.1% (w/v) TNBSA solution for a two-hour reaction at 37°C. A 10% SDS solution was added, followed by the addition of HCl solution (1 N) to stop the reaction. The UV absorbance (335 nm) was measured with a FLUOstar Omega microplate reader (BMG LABTECH). The numbers of the primary reactive amines were calculated by using the calibration curve of 5-amino-1-pentanol.

### Ab affinity checked by surface plasmon resonance (SPR)

SPR experiments were likewise performed as previously described 33. All assays were performed using a CM5 biosensor chip captured with human PKM2 Ag (20 µg/mL, sodium acetate buffer, pH=5). After several running procedures, equilibrium dissociation constants (KDs) were measured by injecting serial dilutions of the Abs at a flow rate of 20 μL/min for 120 s of binding, and 300 s of dissociation. The binding affinity (Kd) was determined by fitting the binding data to a 1:1 kinetic binding model in Biacore T200 (GE Healthcare).

### Ag binding assessed by ELISA

Human PKM2 Ag-coated plates were washed with PBST, blocked with 10% nonfat milk and incubated with the indicated concentrations of Abs for one hour at 37°C. After washing, goat anti-human IgG(H+L)-HRP solution was added for one hour of incubation, followed by the addition of TMB substrate. The absorbance at 450 nm was read with a FLUOstar Omega microplate reader.

### Cellular uptake checked by FACS and confocal imaging

HCC cells were grown under hypoxia or normoxia. To evaluate the cellular uptake of Abs, EDTA-detached cells were incubated with serum-free medium containing the Abs at 37 °C for 1 hour. After PBS-washing, Goat anti-human IgG(H+L)-RDM was added for 1 hour incubation followed by PBS-washing, counting by flow-cytometry, and data were analyzed with FlowJo software. In addition, the attached cells were incubated with Ab-containing serum-free medium at 37 °C for 1 hour, rinsed twice with PBS/NaCl (1 M), fixed with 4% PFA, and blocked with FBS, followed by staining with anti-human IgG(H+L)-RDM and Hoechst 33342 at 4 °C overnight. Cell imaging was conducted with an Olympus FV1000 confocal microscope.

During the confocal imaging process, 30 cells were recorded in each group. In FACS measurements, 10,000 cells were recorded per sample, with triplicate samples per treatment for each experiment.

### Multicellular spheroid binding assay

Multicellular tumor spheroids (MCTSs) were prepared as previously described 29. Briefly, agarose solution (1.5%) was autoclaved, aliquoted and stored at 4 °C. After redissolving by heating, the plate wells were coated with agarose solution, followed by UV-irradiation for sterilization and solidification. A HCC cell suspension (1×10^4^ cells/well) was added to agarose-coated wells and cultured for two weeks to form MCTSs. To evaluate the binding and penetration of the Abs, cultured MCTSs were exposed to Cy7-labeled Abs (400 µM; hIgG as a control) overnight. After washing with PBST buffer, the fluorescence from the MCTSs was visualized via confocal microscopy in z-stack imaging mode.

### Antibody-dependent cellular cytotoxicity (ADCC) assay

ADCC was assayed as previously described 34, 35. Human PBMCs and HCC cells were used as effectors and targets, respectively. Different Abs with a dose-gradient (0.001-10 µg/mL) were incubated with the cells (1x10^4^ per well) for 30 min at 37 °C, after which the cells were incubated at a fixed effector/target ratio (25:1) for 48 hours. A LDH cytotoxicity kit was used to analyze cell viability. Treatment without PBMCs was used as the NC group. ADCC activity was quantified using the equation: % ADCC=(C-E)/Cx100%. E is the final OD value of the experimental groups, and C is the final OD value of the corresponding NC groups. All assays were performed in triplicate, and the percentage of ADCC was plotted against the concentration of corresponding Ab.

### Complement-dependent cytotoxicity (CDC) assay

CDC was assayed as previously described 36. HCC cells (target) were incubated with different Abs in a dose-gradient (0.001-10 µg/mL) for one hour at 37 °C. Fresh human serum from health examiners was used as a complement source and added to the culture mixture (15% serum). After six hours, cell viability was assessed using a LDH assay kit. Heat-inactivated serum was used as a negative control. All assays were conducted in triplicate, and the percentage of CDC was plotted against the concentration of the corresponding Ab.

### *In vitro* cytotoxicity and apoptosis assay

HCC cells (5x10^3^/well) grown under normoxic and hypoxic conditions were starved in serum-free medium for 12 hours. Different Abs (IgG, H103, HAP103 and act-HAP103) were added along a dose-gradient (0.001-1 µg/mL) for 24 hours of incubation. Cell viability and apoptosis were determined by using a CCK-8 kit and an Annexin V-PI kit, respectively, according to the manufacturer's instructions.

### *In vivo* and *ex vivo* imaging in a xenograft mouse model

HCC cells (Huh7, 9×10^6^) were subcutaneously transplanted into 6-week-old female NOD/SCID mice. Tumor growth was monitored once every two days by measuring the diameter of tumors with a caliper, and tumor volumes were calculated according to the formula: Volume = (D × d^2^/2), where D is the longest axis of the tumor and d is the shortest length of a prolate ellipse. When the tumor volume reached approximately 0.4 cm in diameter, the mice were randomly allocated to 6 treatment groups (n=12/group) and intravenously (i.v.) injected with Cy7-labeled Abs (5 mg/kg) combined with TH-302 (50 mg/kg) in PBS. Mice were imaged under anesthesia by an IVIS Lumina II *in vivo* imaging system (Xenogen) at the indicated time points. The fluorescence intensity of the images is reported as photons per second per centimeter squared per steradian (p/s/cm^2^/sr).

For *ex vivo* imaging, Cy7-labeled Abs (i.v., 72 h before euthanization), FITC-pimoniazole (i.p., 60 mg/kg, 30 min before euthanization), and Hoechst 33342 (i.v., 1 mg/kg, 5 min before euthanization) were sequentially injected into half of the mice at the designated time points. After the mice were sacrificed and the heart, liver, spleen, lung, kidney, stomach and HCC were dissected, the fluorescence signals from the organs and tumors were examined and quantified as described above.

### Immunofluorescence imaging on frozen tissue section

Tumor and organ tissues were embedded in O.C.T. freezing medium (Sakura), snap frozen and stored at -80 °C. Tissue sections (10 µm) were first fixed with ice-cold acetone, rehydrated with PBS, blocked with 5% BSA-PBS, double-stained with anti-Hif-1α (1:200) and anti-CD31 (1:200) Abs overnight, and finally incubated with goat anti-rabbit IgG-AF488 (1:1000). Multiple fluorescence signals were visualized under a fluorescence microscope (Olympus BX51).

### *In vivo* continuous targeted therapy and toxicity study

To study the long-lasting therapeutic response, Huh7 cells were xenografted into mice, which were continuously injected seven times with naked Abs (5 mg/kg) combined with TH-302 (50 mg/kg) (n=8/group). Tumor growth in surviving mice was monitored by caliper measurements once a week. Mice were euthanized when the tumor size reached a diameter of 1.5 cm, tumors ulcerated, or there was any sign of mouse distress. One week after the last dose of Ab therapy, the mice were euthanized, and the heart, liver, spleen, lung, stomach and HCC tumors were surgically removed, weighed, and fixed in 10% PFA for 48 hours. Then, the fixed tissues were embedded in paraffin, and the sections were subjected to HE and TUNEL staining.

### Blood sample and lymphocyte culture

Human heparinized peripheral blood was collected from health examiners. Peripheral blood mononuclear cells (PBMCs) were isolated with lymphocyte separation medium as described previously 37. The cells were centrifuged for 30 min, collected, resuspended, and incubated with RBC lysis buffer for 15 min. After cell-spinning, the cells were resuspended in PBS, laid over Ficoll and centrifuged for 45 min. The top layer, which contained mostly monocytes, was collected in complete medium and cultured for assays.

### Patient samples and immunohistochemistry (IHC)

Ten fresh surgical specimens from HCC patients were collected from the Cancer Hospital of Tianjin Medical University. Tissue microarrays of multiple human organs (TMAs, X36Mc01) were obtained from Zhong Ke Guang Hua Co., Ltd (China). Mouse organ tissues were derived from xenograft mice. IHC staining was performed as previously reported. 25, 38-40 Briefly, after deparaffinization, rehydration, and Ag-retrieval treatment with sodium citrate buffer (10 mM, pH=6.0) at 120 °C for 5 min, the slides were treated with hydrogen peroxide to quench endogenous peroxidase activity, blocked with goat serum, and incubated with primary Abs (0.1 mg/mL) overnight at 4°C. Goat anti-human IgG-HRP was used as a secondary Ab, and normal rabbit serum was used as a NC. IHC staining was performed with an EnvisionTM system (Dako, USA).

### Statistical analysis

All the results are presented as the Mean ± standard error. Statistical analyses were performed with GraphPad Prism using One--way ANOVA, Two-way ANOVA, or Student's t-test, as indicated in the legends. A significant difference was defined as *p<0.05 or **p<0.01. Three independent trials were conducted unless otherwise stated.

### Study approval

Animal experiments were approved by the Nankai University Animal Care and Use Committee (2021-SYDWLL-000479). Human blood samples were obtained from health examiners, and surgical specimens were obtained from HCC patients (informed consent was obtained from the Review Board of Nankai University, NKUIRB2021043).

## Results

### Design and development of hypoxia-activated anti-PKM2 Ab HAP103

**Figure 1:**
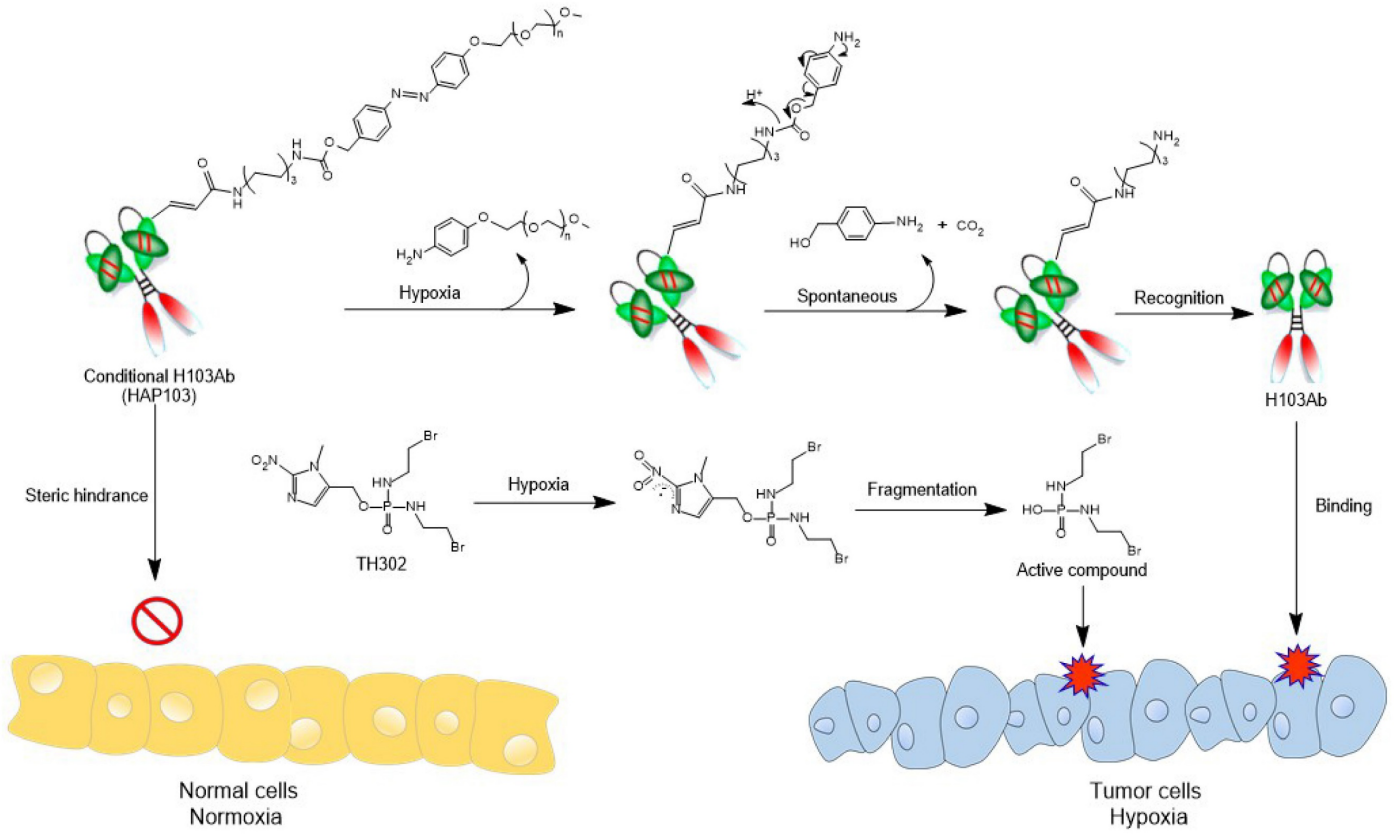
Schematic diagram illustrating the 'double kill' effect of a hypoxia-activated HAP103 Ab combined with a hypoxia-activated TH-302 prodrug.

Hypoxia-activated Ab HAP103 was developed by conjugating the full-length Ab H103 with PEG5k-azo ester. This conditional Ab comprises a target-recognized unit controlled by a caging moiety such that tethered PEG5k-azo sterically blocks the binding between H103 Ab and PKM2 Ag. Once azo bonds are reduced in a hypoxic microenvironment, a self-immolative reaction releases PEG5k, and conditional HAP103 Ab is allowed to internalize and bind with PKM2 in hypoxic cells, thus eradicating on-target off-tumor toxicity before targeting hypoxic HCC. This design provides the basis for synergistic therapy by combining this Ab with HAP TH-302 (Fig. 1).

**Figure 2:**
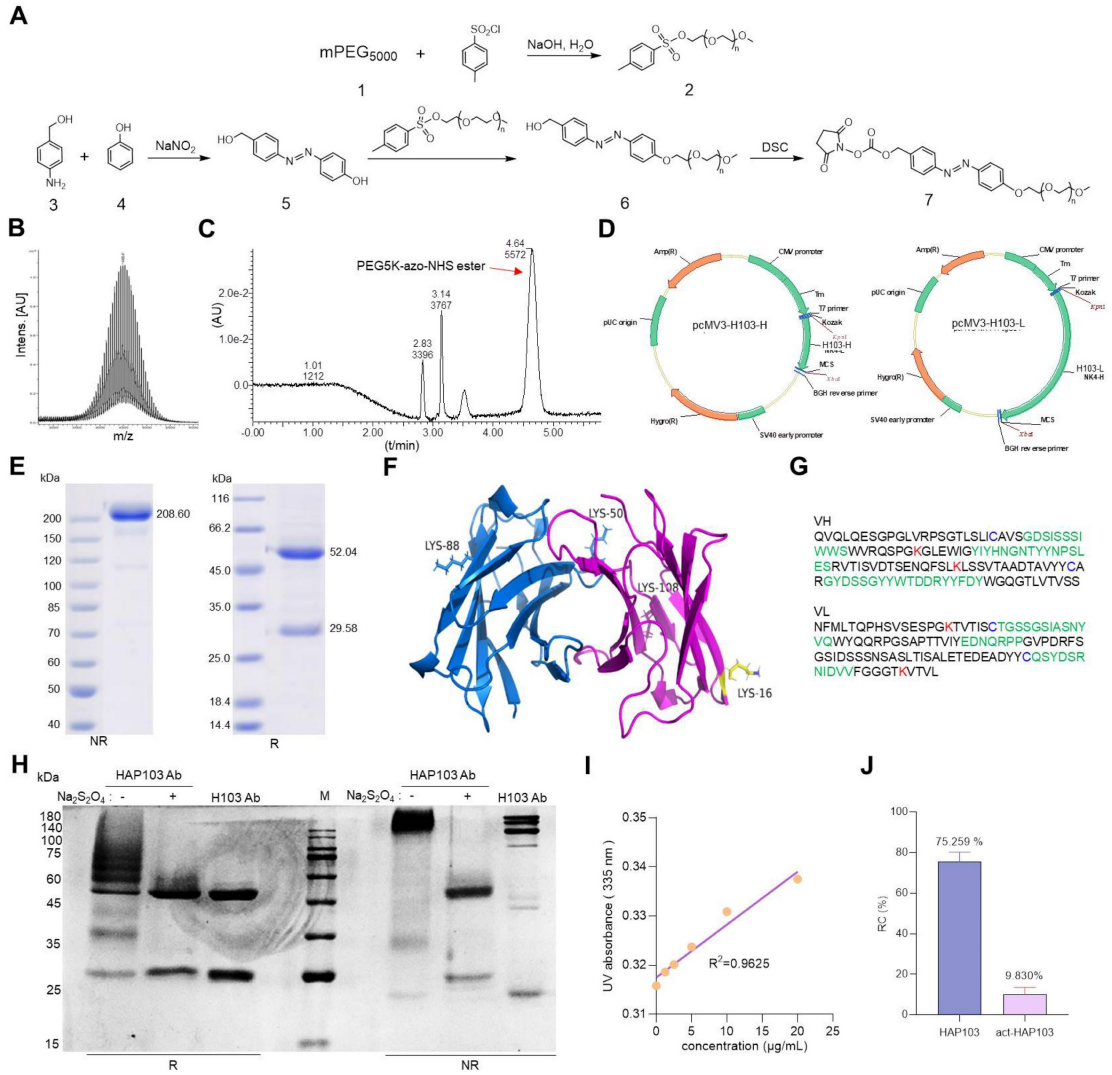
Synthesis of amine-reactive PEG5K-azo and creation of HAP103 Ab. (**A**) Synthesis scheme of amine-reactive PEG5K-azo ester. Compounds #1-7 were characterized as shown in the supplementary file. (**B**) Mass spectrum of PEG5K-azo ester (compound #7). (**C**) HPLC absorbance spectrum of synthesized PEG5K-azo ester (compound #7). (**D**) Schematic map of recombinant expression vectors containing H103Ab-H and H103Ab-L chain genes. (**E**) SDS-PAGE analysis of fine-purified H103Ab samples after the SEC-HPLC (size-exclusion high performance liquid chromatograph) analysis. R: reduced sample. NR: nonreduced sample. (**F**) The VL (magenta) and VH (blue) structures of H103Ab were predicted by PIGS programs, and surface accessible lysine residues are shown. (**G**) VH and VL sequence features of H103Ab were rendered to show the key amino acids contributing to its idiotope. CDR: green characters. Lysine (K): red characters. Cysteine (C): blue characters. (**H**) SDS-PAGE analysis of HAP103 Ab with or without Na_2_S_2_O_4_ pre-treatment. Ab samples were loaded under nonreducing and reducing conditions. (**I**) A calibration curve of the TNBSA assay was constructed to quantify the number of primary amino groups within the H103 Ab. 5-Amino-1-pentanol was used as a standard regent. (**J**) Conjugation ratio (R_C_) of PEG5K to HAP103 and activated HAP103 cleaved by Na_2_S_2_O_4_. The RC was defined as the percentage of PEG5K conjugated to accessible amino groups.

To develop conditional HAP103 Ab, an amine-reactive PEG5k-azo ester was synthesized as a caging moiety following previous methods with some modification 28, 29, 41 (Fig. 2A-C, Supplementary Fig. S1). To increase the Ab expression level, the first five amino acids in the VH of H103 were replaced by those in the germline gene (IGHV4-4*02), and the recombinant Ab was synthesized via total codon optimization (Supplementary Table. S1, SinoBiological Co. Ltd). Then, human H103 Ab construct was expressed in the HEK293 cell line, which generates the full-length form of IgG1 with targeting activity (Fig. 2D). After serum-free cell culture in a bioreactor, the secreted human H103 Ab was finely purified (Fig. 2E). Structural modeling of the H103 Ab revealed that four lysine residues required for PEGylation were surface accessible in the VL and VH domains (Fig. 2F-G, Supplementary Table S2). After conjugating H103 Ab with the as-prepared PEG5k-azo ester, reducing and nonreducing gel electrophoresis showed that HAP103 Ab with increased molecular weight was generated (Fig. 2H). To mimic the hypoxia-response of HAP103 Ab, reconversion reactions were conducted by treating HAP103 Ab with Na_2_S_2_O_4_, which can cleave azo bonds 28, 29. Gel electrophoresis showed that, after Na_2_S_2_O_4_ treatment, the bands corresponding to HAP103 Ab disappeared, whereas the bands corresponding to parental H103 Ab reappeared (Fig. 2H). To determine the conjugation efficiency, TNBSA assays were performed, and an average of 75% of the accessible lysine residues were conjugated with PEG5k-azo. Again, Na_2_S_2_O_4_ treatment abolished the conjugation (Fig. 2I-J). These results showed that PEG-azo-conjugation endows HAP103 Ab with hypoxia-activating properties.

### HAP103 Ab conditionally targets PKM2 Ag in hypoxic cancer cells and tissues

**Figure 3:**
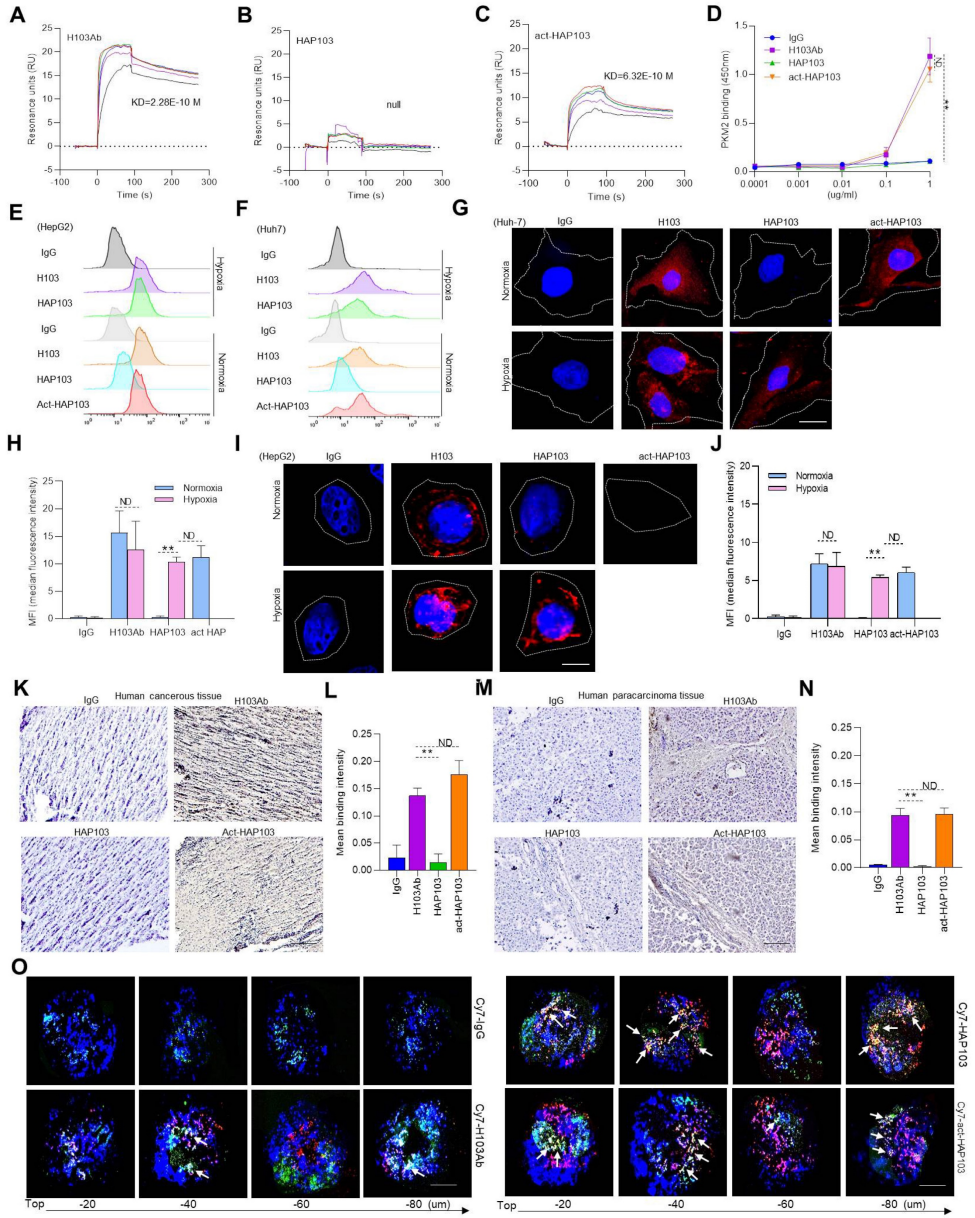
*In vitro* binding features of HAP103 Ab. (**A**, **B**, **C**) SPR-determining the affinity of H103 (A), HAP103 (B) and Na_2_S_2_O_4_-cleaved act-HAP103 (C) Abs for the purified PKM2 antigen. (**D**) ELISA analysis of different Abs bound to PKM2 antigen. n=3. Mean±SD, **P < 0.01 vs. H103 Ab. ND: no significant difference. One-way ANOVA. (**E, F**) Representative flow cytometry analysis of different Abs internalized into HepG2 (E) and Huh7 (F) cells cultured under hypoxia and normoxia. hIgG was used as a control, and representative data are shown. (**G, I**) Representative confocal imaging of Abs-internalized Huh7 (G) and HepG2 (I) cells under hypoxia and normoxia. (**H, J**) Confocal microscopy was used to determine the uptake of Ab into Huh7 (G) and HepG2 (I) cells, and the results were quantified and compared with those obtained with hIgG. n=30. Mean±SD, **P < 0.01 vs. normoxia. ND: no significant difference vs. normoxia, or act-HAP103 vs. HAP103 Ab. Student's t-test. (**K, M**) IHC staining of cancer (K) and paracarcinomatous tissue (M) samples from late-stage liver cancer patients. Scale bar, 200 μm. (**L, N**) Abs bound to the tissues was quantified. hIgG was used as a control. n=10. Mean±SD, **P < 0.01 vs. H103 Ab. ND: no significant difference. One-way ANOVA. (**O**) Confocal imaging of different Abs bound and internalized into Huh7 MCTSs. The fluorescence signals from Cy7-labeled Abs and AF488-directed anti-Hif1a Abs were collected at different levels from the top to the middle of the spheroids on z-axis. Scale bar=200 μm.

Then, we tested the *in vitro* binding performance of HAP103 Ab to purified PKM2 Ag. SPR assays showed that the affinity of HAP103 Ab was completely undetectable compared to that of parental H103 Ab. This attenuation was reversed when HAP103 Ab was cleaved by Na_2_S_2_O_4_ (Fig. 3A-C). ELISA showed that, compared with that of parental H103 Ab, the binding of PKM2 to HAP103 Ab was abolished even at high-concentrations. However, act-HAP103 Ab completely recovered the binding to PKM2 (Fig. 3D). Next, we tested the cellular uptake of the HAP103 Ab. Flow-cytometry analysis revealed that HAP103 Ab did not internalize and recognize the expressed PKM2 Ag in normoxia-cultured HCC cells. However, act-HAP103 Ab recovered the targeting and internalization to HCC cells. Such activation-triggered targeting recovery was also observed when HAP103 Ab was added to hypoxia-cultured HCC cells (Fig. 3E-F, Supplementary Fig. S2). Confocal imaging further revealed that the uptake of HAP103 Ab by normoxic HCC cells decreased. In contrast, the uptake of Abs was very close to that of H103 Ab when HAP103 was added under hypoxic conditions. Additionally, the act-HAP103 Ab strongly internalized and targeted normoxic HCC cells (Fig. 3G-J). These results indicate that PEG-azo_-_conjugation could cause steric hindrance to prevent HAP103 Ab from targeting PKM2 under normoxia; but release such hindrance when hypoxia occurs.

The targeting of conditional HAP103 Ab to HCC tissues was further checked *ex vivo*. Compared with parental H103 Ab, HAP103 Ab bound weakly to HCC tissues. However, once cleaved by Na_2_S_2_O_4_, the act-HAP103 Ab showed strong binding to cancer tissue (Fig. 3K-N). The MCTS has been proposed as a model for evaluating hypoxia-activated drug delivery 29. We prepared Huh7 MCTSs as models to assess whether HAP103 Ab could recognize PKM2 Ag in hypoxic HCC. Huh7 MCTSs were incubated with Cy7-labeled HAP103, act-HAP103, H103 Abs and control hIgG. The hypoxic region inside the tumor tissue was targeted by the anti-Hif1a Ab developed from the AF488-labeled 2^nd^ Ab. The Ab-directed fluorescence signals in MCTSs were recorded via confocal imaging. In routine cultured MCTSs, exposure to Cy7-labeled H103 Ab resulted in a certain extent of penetrating fluorescence signal. However, only a little colocalized fluorescence signals were observed in hypoxic area. In contrast, strong colocalized fluorescence signals (Cy7 and AF488) were detected in hypoxic area of MCTSs exposed with HAP103 Abs. Notably, MCTSs exposed to act-HAP103 Ab presented more intensive penetrated colocalized fluorescence signal than that of MCTSs treated with H103 Ab (Fig. 3O). This phenomenon was much obvious when hypoxia-cultured MCTS exposed with these Abs (Fig. S3). These results suggest that PEG-azo-conjugation could obstruct H103 Ab binding to PKM2 Ag in HCC tissues, completely removing this targeting before hypoxia-triggered reactivation.

### Activated HAP103 Ab induces potent antitumor and immunity activity *in vitro*

**Figure 4:**
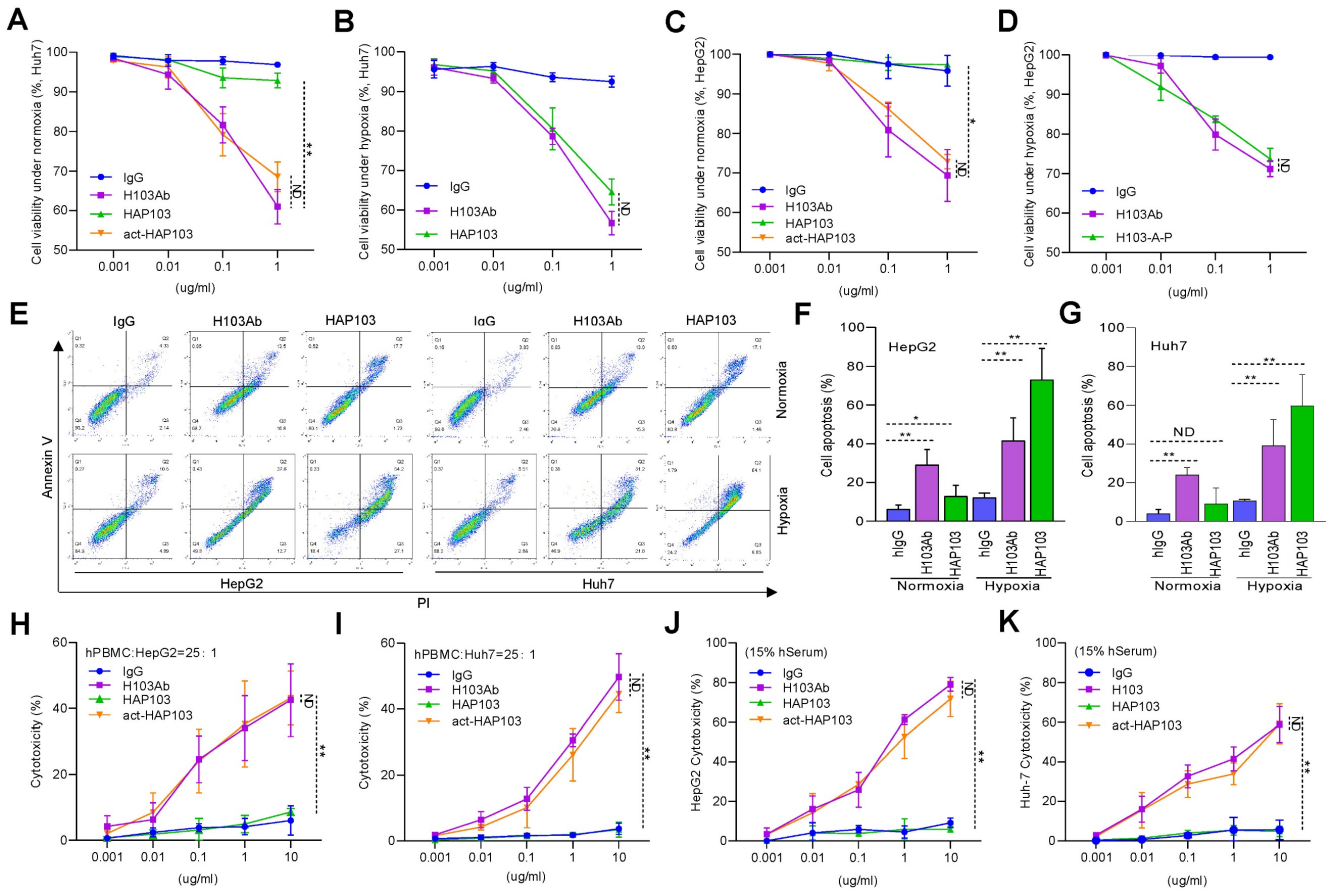
*In vitro* biological function of HAP103 Ab. (**A**, **B**) Proliferation efficiency of normoxic (A) or hypoxic (B) Huh7 cells inhibited by H103, HAP103 and act-HAP103 Abs, as determined by CCK-8 assays. (**C**, **D**) Proliferation efficiency of normoxic (C) or hypoxic (D) HepG2 cells inhibited by different Abs was tested by CCK-8 assays. n=3. Mean±SD, **P < 0.01 vs. H103 Ab. ND: no significant difference. One-way ANOVA. (**E**) Representative images of normoxic and hypoxic cell apoptosis triggered by different Abs. (**F**, **G**) Analysis of Abs-induced total apoptosis in HepG2 (F) and Huh7 (G) cells cultured under hypoxia or normoxia. A statistical difference compared with that of hIgG was demonstrated. n=3. Mean±SD, **P < 0.01 vs. hIgG. ND: no significant difference. Two-way ANOVA. (**H**, **I**) ADCC activity mediated by H103, HAP103 and act-HAP103 Abs in LDH release assays. hPBMCs were used as effectors, and HepG2 (H) or Huh7 (I) cells were used as targets in fixed effector/target ratio (25:1). (**J**, **K**) CDC activity mediated by different Abs in HepG2 (J) or Huh7 (K) cells. n=3. Mean±SD, **P < 0.01 vs. H103 Ab. ND: no significant difference. One-way ANOVA.

PKM2 is a key rate-limiting glycolysis enzyme overexpressed in HCC and represents a new potential therapeutic target 42-44. To test whether the HAP103 Ab could block PKM2-mediated malignant phenotypes, we further analyzed its antitumor activity at the cellular level. CCK-8 assays showed that normoxic HCC cells treated with HAP103 Ab proliferated normally, whereas HAP103 Ab treatment markedly inhibited cellular proliferation under hypoxic conditions. This impaired proliferation efficiency could be mimicked by incubating normoxic cells with act-HAP Ab (Fig. 4A-D). Because PKM2-targeting can induce apoptosis in cancer cells 45, 46, we further compared the degree of apoptosis triggered by these Abs. Annexin V-PI assays showed that H103 Ab treatment induced a moderate apoptosis in HCC cells both under normoxia and under hypoxia. However, HAP103 Ab induced notable apoptosis in hypoxic HCC cells, which was much greater than that induced by parental H103 Ab (Fig. 4E-G). These *in vitro* results suggest that hypoxia-activated HAP103 Ab induces enhanced antitumor activity by inhibiting cell proliferation and triggering increased apoptosis in HCC cells.

As the effector functions of Fc fragment are essential for Ab therapy, we further tested whether the conditional HAP103 Ab retained its effector function. The ADCC activity of Abs was first compared. hPBMCs were used as effectors, and HCC cells were used as targets. LDH release assays showed that HAP103 Ab lost its ADCC activity even when the effector/target ratio was increased to 25:1. Excitingly, this impaired ADCC activity was completely reversed when the HAP103 Ab was activated by Na_2_S_2_O_4_ treatment (Fig. 4H-I). We next checked whether the CDC activity of conditional Ab was altered after conjugation with PEG5k. When fresh hSerum was used as the complement source, LDH release assays showed that the CDC activity of HAP103 Ab lost. In contrast, the Na_2_S_2_O_4_-activated HAP103 Ab completely restored its CDC activity (Fig. 4J-K). These *in vitro* results suggest that act-HAP103 Ab can induce a potent immunological response.

### Activated HAP103 Ab exerts selective targeting and potent therapeutic efficacy *in vivo*

**Figure 5:**
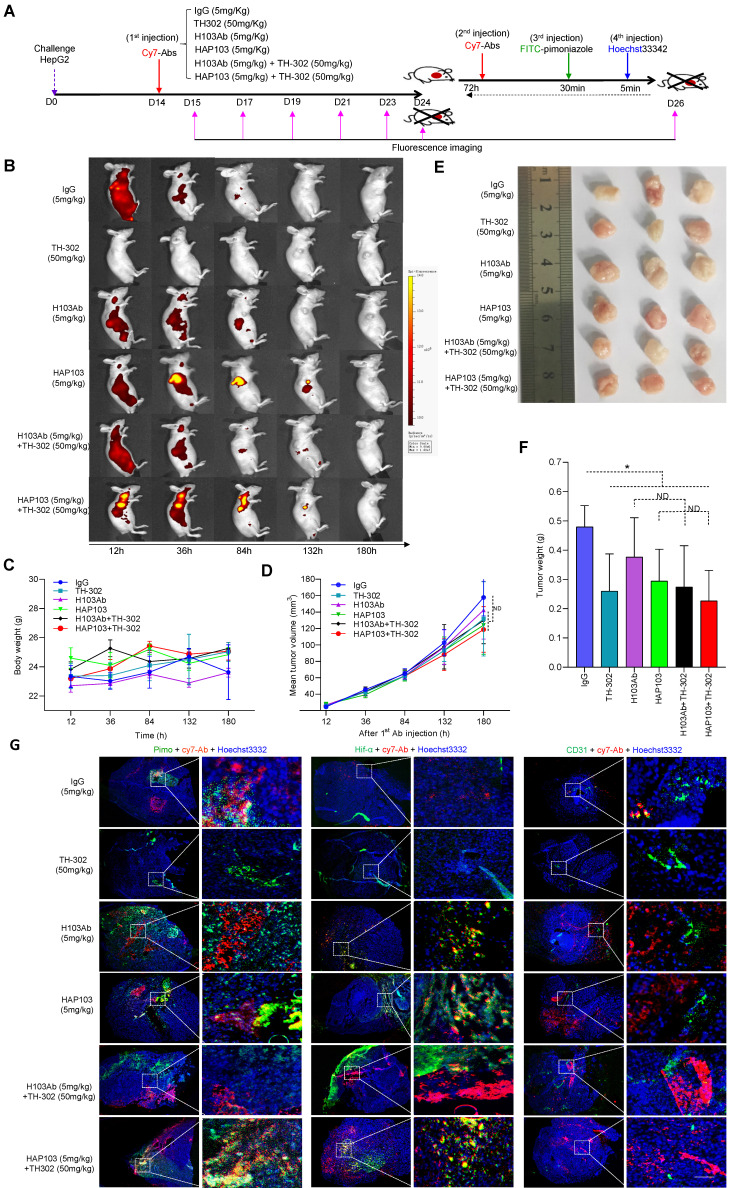
*In vivo* selective imaging and targeting of liver cancer cells with HAP103 Ab. (**A**) Schedule of Cy7-labeled Ab imaging and targeting combined with TH-302 in human HCC-xenografted mice model. To check the targeted selectivity, half of the mice received different Abs (H103Ab, HAP103, or hIgG as a control), TH-302, or combined injections only once, followed by dynamic fluorescence imaging. To test hypoxia-targeting, half of the mice received a 2^nd^ injection followed by pimoniazole and Hoechst 33342 injection for tissue section imaging. (**B**) Representative *in vivo* fluorescence imaging of tumor-bearing mice injected with Cy7-labeled Abs and TH-302 through the tail vein. (**C**) Body weight curves of xenografted mice treated with different Abs (measured 24 hours after drug administration). n=6. (**D**) Growth curve of liver cancer volume after treatment with the Abs, TH-302, or the combination of two kinds of agents at different doses. n=6. Mean±SD, **P < 0.01 vs. hIgG. Student's t-test. (**E**) Representative images of excised tumors (half mice) from different groups are shown at 24 days. (**F**) Weights of excised tumors from the different treatment groups. n=6. Mean±SD, **P < 0.01 vs. hIgG. ND: no significant difference. Student's t-test. (**G**) Overlays of multiple digital fluorescence images of Huh7-derived HCC tissue from mice injected with Cy7-labeled Abs (red. 72 hours before euthanization) and FITC-labeled pimonidazole (green. 30 min before euthanization), or perfusion with Hoechst 33342 (blue, 5 min before euthanization), or colocalization with blood vessel makers (green, anti-CD31 Ab staining). Representative *ex vivo* fluorescence signals from liver cancer tissues from treated mice are shown.

**Figure 6:**
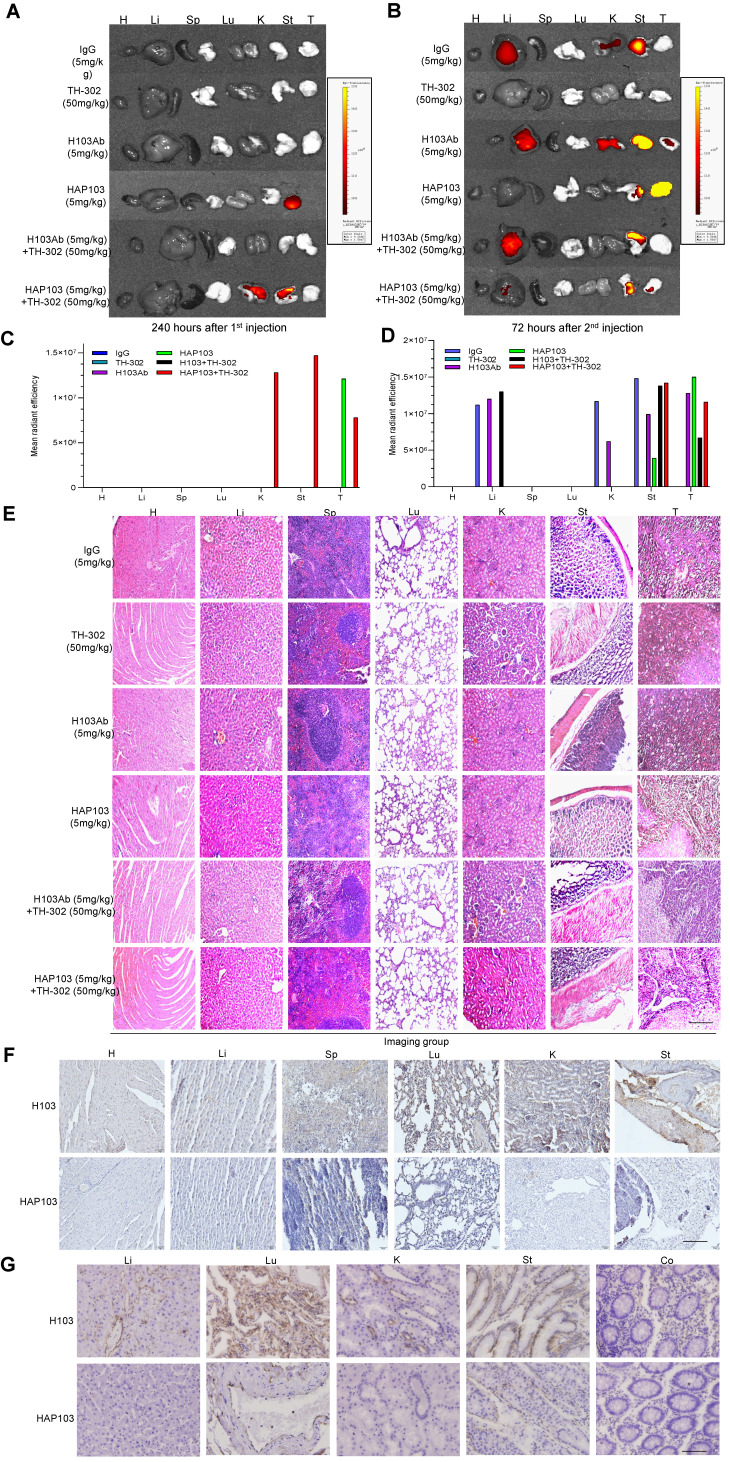
*Ex vivo* identification of the targeting-associated toxicity of HAP103 Ab. (**A, B**) Representative fluorescence signals from excised organs and liver cancer tissues from mice injected with Cy7-labeled Abs and TH-302 through the tail vein. Heart (H), liver (Li), spleen (Sp), lung (Lu), kidney (K), stomach (St) and tumor (HCC) tissues. (**C, D**) Quantification of Cy7 fluorescence signals in main organs and liver cancer tissues. The average values from three mice are shown. (**E**) Representative H&E staining of main organs and liver cancer tissues from mice subjected to different treatments. (**F, G**) Representative IHC staining of normal tissues from multiple human organs (tissue microarray, F) and xenograft mice (G) using H103 and HAP103 Ab Abs at matching concentrations. Colon (Co).

To test whether HAP103 Ab could be used for tumor imaging *in vivo*, we systematically analyzed its ability to target tumors in NOD/SCID mice xenografted with HCC cells (cell derived xenograft, CDX) (Fig. 5A). As early as 12 hours after the injection of Cy7-labeled Abs through the tail vein, the fluorescence signals from the Abs were diffusely gathered at the HCC sites. However, only the accumulation of HAP103 Ab was relatively exclusive (Fig. 5B). The tumor-targeted fluorescence signals were contrastively maintained over five days post injection of the Cy7-labeled HAP103 Ab. In contrast, accumulated fluorescence signals were not visualized in HCCs post injected with Cy7-labeled H103 Ab, and the dispersal fluorescence signals disappeared at approximately 84 hours post injection (Fig. 5B). To compare the targeting distribution of Abs, half of mice in each group were sacrificed at 10 days after the 1^st^ injection, the other half were sacrificed at 72 hours after the 2^nd^ injection combined with pimoniazole, and tumors and main organs were collected for fluorescence imaging. *Ex vivo* imaging showed that the Cy7-labeled HAP103 Ab selectively accumulated in xenografted HCC tumors, whereas H103 Ab nonspecifically bound to the stomach, kidney and liver in mice (Fig. 6A-D). This sharp-contrasted targeting profile was less observed when the time points of *ex vivo* imaging were extended. In addition, IHC staining indicated that HAP103 Ab barely bound to mouse organal tissues, whereas parent H103 Ab distinctly cross-reacted with normal tissues (Fig. 6F, G). The targeting ability of the conditional Ab was further analyzed by costaining the excised HCC tissues with Abs against Hif-1α, microvascular marker CD31 and hypoxia marker pimonidazole. The signals from Cy7-labeled HAP103 Ab, but not from Cy7-labeled H103 Ab, were strongly colocalized with the signals from FITC-labeled pimonidazole and AF488-indicated Hif-1α, but not overlapped with AF488-indicated CD31 (Fig. 5G). These results indicate that conditional HAP103 Ab could be selectively activated* in vivo* and recognize its target PKM2 in hypoxic HCC tissues, whereas the parental H103 Ab lacks such potential.

**Figure 7:**
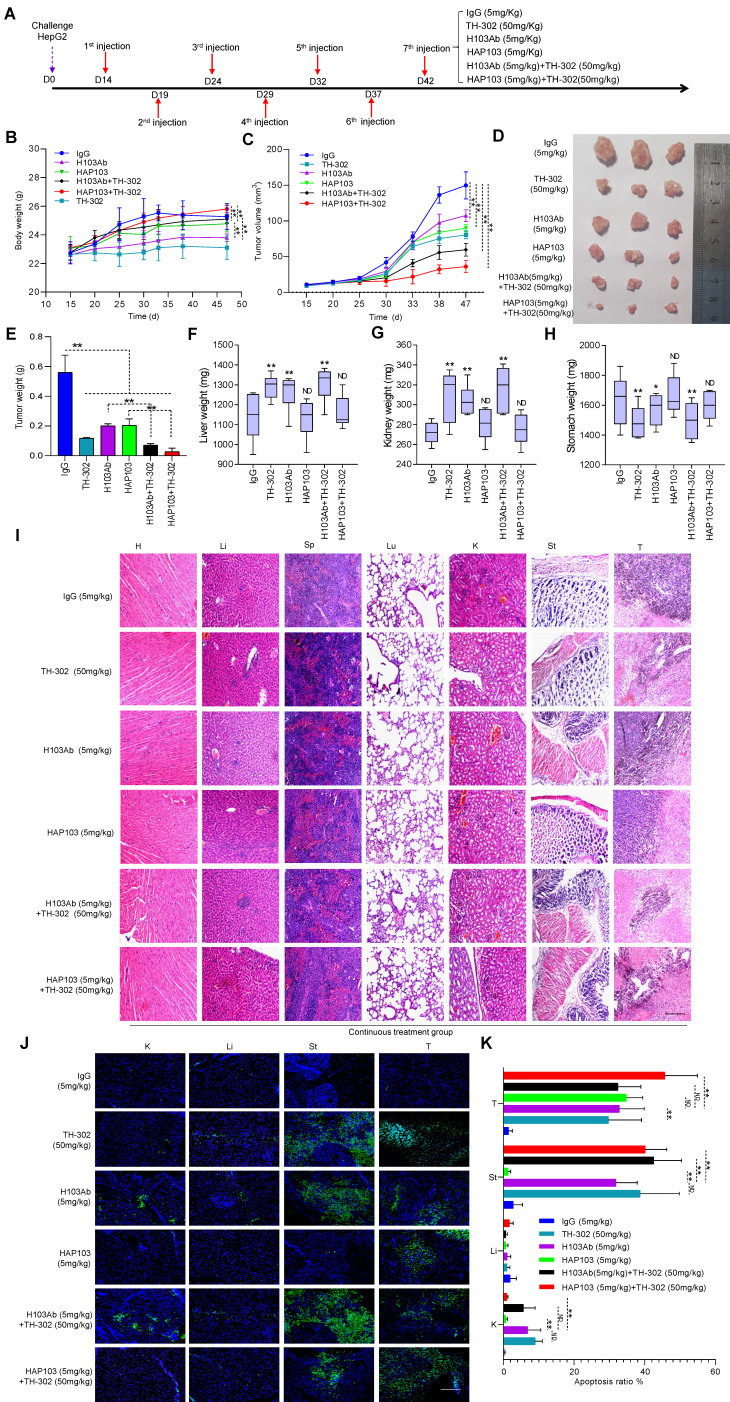
Hypoxia-targeting therapy for liver cancer via the combination of selectivity-improved HAP103 Ab and prodrug TH-302. (**A**) Schedule of continuous targeted therapy with the Abs (hIgG, H103, or HAP103) combined with TH-302 in HCC-xenografted mice. **(B)** Body weight curves of xenografted mice treated with different Abs (measured beginning on the 15^th^ day postadministration of Huh7 cells. n=6. Mean±SD, **P < 0.01 vs. hIgG or H103 Ab. Student's t-test. (**C**) Growth curves of liver cancer volume affected by Abs, TH-302, or the combination treatment at different doses. n=6. Mean±SD, **P < 0.01 vs. hIgG. One-way ANOVA. (**D**) Representative photographs of excised tumors (half mice) from different groups are shown at 47 days. (**E**) Weights of excised tumors from different treatment groups. n=6. Mean±SD, **P < 0.01 vs. hIgG. or **P < 0.01 vs. monotherapy. Student's t-test. (**F**, **G**, **H**) Liver, kidney and stomach weights of mice treated with hIgG, H103, TH-302, HAP103, or different combinations of these agents. n=6. Mean±SD, **P < 0.01 vs. hIgG. ND: no significant difference. One-way ANOVA. (**I**) Representative H&E staining of main organ tissues from mice subjected to different treatments. (**J**) Representative TUNEL staining demonstrates the degree of apoptosis in main organs and xenografted HCC tissues after different treatments. Scale bar=500 µm. (**K**) Apoptosis signals from distinct tissues were quantified and analyzed. n=6. Mean±SD, **P < 0.01 vs. H103Ab. One-way ANOVA.

To test the therapeutic potential of conditional Ab combined with TH-302, the tumor-killing efficacy of the combination therapy in xenograft models was further analyzed. Unexpectedly, one time monotherapy with HAP103 Ab did not attenuate the progression of HCC, in terms of tumor volume or weight, compared with that induced by H103 Ab (Fig. 5D-F). When TH-302 was combined with any single treatment, these outcomes did not improve. Furthermore, continuous weekly injections of HAP103 Ab significantly inhibited tumor growth. In contrast, continuous injections of H103 Ab only induced HCC regression in some mice. Notably, continuous injection of HAP103 Ab combined with TH-302 almost completely inhibited tumor growth, and such a combination exhibited better tumor-inhibiting efficacy than that by H103 Ab combined with TH-302 (Fig. 7C-D). TUNEL staining assays showed that treatment with HAP103 Ab significantly induced apoptosis in HCC tissues, which was consistent with the effect of H103 Ab (Fig. 7J-K). These results suggest that targeting therapy with conditional HAP103 Ab induces potent antitumor activity *in vivo*, that is superior to that induced by parental H103 Ab.

### Activated HAP103 Ab has improved biosafety without systemic toxicity *in vivo*

Finally, to evaluate the safety of conditional HAP103 Ab, the toxicity profiles of different Abs in mice were compared. No significant body weight loss was observed upon treatment with HAP103 Ab. In contrast, mice treated with H103 Ab, especially TH-302, experienced significant weight loss (Fig. 7B). Checking the surgically resected organs from euthanized mice showed that treatment with H103 Ab or TH-302 resulted in gross enlargement of the liver and kidney and evident kidney atrophy, gastratrophia and stomach injury. In contrast, treatment with HAP103 Ab or HAP103 Ab combined with TH-302 barely led to hepatomegaly, kidney atrophy or gastratrophia, as demonstrated by organ weight (Fig. 7F-H). H&E staining of major organs showed that treatment with conditional HAP103 Ab alone or in combination with TH-302 caused less damage to major organs. In contrast, mono-treatment with H103 Ab or TH-302 caused obvious histological and morphological changes in kidney and liver tissues. In some of the mice, H103 Ab or TH-302 treatment caused significant hepatomegaly, hepatic venous congestion and nephrorrhagia (Fig. 6E. Fig. 7I). These results indicate that the augmented toxicity in these organs was due to nonspecific targeting by H103 Ab, and hypoxia-activated HAP103 Ab could considerably alleviate systemic toxicity while maintaining high therapeutic efficacy.

## Discussion

Although numerous studies have linked hypoxia to malignant phenotypes and poor prognosis, targeting hypoxia has not yet become a clinical standard for HCC treatment. This may be due to cognitive mistake related to HCC. Although the liver is a hypervascular organ, HCC is actually a hypoxic malignancy (Supplementary Fig. S4). This difference can be attributed to the fact that highly proliferating HCC cells induce local hypoxia when they are far from blood vessels. Second, HCC generally develops through cirrhosis, which causes fibrogenesis and leads to hypoxia. Third, interventional therapies, such as transcatheter arterial embolization (TAE) and transcatheter arterial chemoembolization (TACE), always cause catastrophic hypoxia 47. This hypoxic microenvironment can be capitalized to create HCC-selective targeting approaches by using bioreductive blockers or HAPs 48-50. Pervious combination studies have used chemotherapeutics or therapeutic Abs to target oxygenated tumor cells and HAPs to target hypoxic compartments 5, 9, 12, 16-23, 51-54. However, the on-target off-tumor toxicity of Ab sharply limits the clinical efficacy of combined treatments 24, 55-60. Here, we demonstrated that the generation of a hypoxia-activated anti-PKM2 Ab can eliminate the side effects of parent Ab and improve its efficiency in selectively targeting HCC cells (Fig. 5-6). Notably, the combination of hypoxia-activated TH-302 with a hypoxia-activated anti-PKM2 Ab is a novel and powerful way to treat HCC through a synergistic “double kill” effect (Fig. 7).

PKM2 is well documented to be overexpressed and to play a crucial role in the aerobic glycolysis pathway in cancer cells 61, 62. Some researchers have argued that PKM2, a cytoplasmic enzyme, is not expected to localize to cell surface. In hypoxic cancers, quite a fraction of PKM2 Ag can translocate to the cell surface. First, a specific signal can direct truncated forms of pyruvate kinase trafficking to the cell surface 63. Second, many adhesion molecules, such as CD44, were shown to bind with PKM2 on the cancer stem cell surface 64. Third, previous works, including ours, have shown that apoptotic or hypoxic stress induce the translocation of PKM2 to the nucleus and cell surface in several cancers 25, 65, 66. Fourth, ethanol or hypoxia increases the expression of PKM2, which is frequently observed in exosomes and ectosomes secreted from the cancer cell surface 67, 68. Moreover, our current and previous studies both indicate that hypoxia induces increased PKM2 expression in HCC 25 (Supplementary Fig. S5). These findings mirror well with clinical observations that elevated PKM2 is involved in the treatment resistance of HCC patients receiving TACE 69. Four possible mechanisms underlie PKM2 overexpression in hypoxic cancer cells. First, hypoxia mediates PKM2 transcription by enhancing HIF1a DNA-binding activity and recruiting p300 to the HRE in intron I of PKM2 gene 70. Second, PKM2 can interact with Hif1a and promote the transactivation of Hif1a downstream genes. Because this interaction is mediated by PHD3-dependent PKM2 hydroxylation and because both PKM2 and PHD3 are target genes of Hif1a, the resulting positive feedback loop maintains Hif1a activity and PKM2 overexpression 71. Third, hypoxia can transcriptionally induce HIF1α to promote YTHDF1 expression, which enhances PKM2 expression and increases tumorigenesis 72. Fourth, hypoxia-induced LncRNA DACT3-AS1 upregulates PKM2 expression to promote HCC metastasis through the HDAC2/FOXA3 pathway 73. These findings, as evidenced by ours and others 74, solidly indicate that hypoxia-triggered overexpression and translocation of PKM2 in HCC cells is a potential target for Ab-based therapeutics.

Hypoxia-activated designs have been developed for creating cancer-selective prodrugs 49, 51, 75. Most of these designs involve prodrug activation by utilizing quinones, nitroaromatics and N-oxides as release triggers. Comparatively, only a few studies have adopted azo moieties to design hypoxia-activated prodrugs 26, 76. As a nitroimidazole derivative, azo groups are susceptible to oxygen-dependent destabilization under reducing conditions, which quickly results in the cleavage of linked unit. To establish hypoxia-activated theranostics, some studies have incorporated azo moieties in hypoxia-degradable vehicles to conditionally deliver drug-loaded nanomaterials, such as siRNA-PEG-PEI-DOPE copolymer and Dox-HPMPC nanogel 77, 78. Other studies have adopted azo groups as a scaffolds to conditionally quench imaging reagents including photosensitizer selenorosamine dyes 79, rhodamine derivatives 27, 80 and NIR probes 76, 81, 82. To achieve the hypoxia-induced elimination of drug toxicity, several studies have synthesized azo derivatives and conjugated them to small molecule chemotherapeutics, such as alkylguanine-DNA alkyltransferase inhibitor laromustine 49, 80, DNA alkylating agent benzenediamine 83, doxorubicin and nitrogen mustard 84, 85. However, similar to our proposal (Fig. 1), only two works have designed azo-based conditional delivery of Abs. One study developed hypoxia-activated aptamer probes by conjugation with PEG-azo, which acts as a caging moiety for conditional binding 29. Despite the fact that this study reported a PEGylated conditional Ab, no *in vivo* data support this concept. Another study designed hypoxia-activated Ab-drug conjugates by linking MMAE to an anti-HER2 Ab through p-nitrobenzyl-based and nitroimidazole-based linkers 86. Although remarkable tumor killing was induced by such a 'pro-prodrug' design, there is still certain cytotoxicity derived from hypoxia-triggered MMAE release. Comparatively speaking, our current work, validated by *in vitro* and *in vivo* data (Fig. 2-7), highlights the azo-based conjugation strategy can be convincingly employed to create hypoxia-activated Ab drugs.

The main objective of this study was to establish a safe, tolerable, synergistic treatment for HCC by using hypoxia-activated HAP103 combined with hypoxia-activated prodrug TH-302. For both dosing schedules, hepatic, kidney and gastric toxicity was rarely observed in the combined treatment groups (Fig. 6E. Fig. 7I). This finding is in sharp contrast to that of single treatment with H103 Ab or TH-302, which frequently caused moderate or severe hepatomegaly, kidney atrophy, gastratrophia and stomach injury (Fig. 7F-I). Compared to that of parent H103 Ab plus TH-302, the additive cytotoxic activity but diminished off-target toxicity induced by conditional HAP103 Ab plus TH-302 is appealing. This combinatorial approach completely reduces the harmful side effects. Remarkably, the anti-HCC efficacy of hypoxia-activated prodrug TH-302 was enhanced when TH-302 was combined with hypoxia-activated HAP103 Ab (Fig. 7C-E). This could be attributed to the improved targeting selectivity of anti-PKM2 Ab against HCC, and higher Ab doses should be used in future studies. Although one injection of HAP103 Ab did not result in tumor inhibition (Fig. 5E, D-F), treatments with continuous HAP103 Ab exhibited significant HCC-killing activity even at low doses (5 mg/kg) (Fig. 7C-E). These findings imply that treatment at high doses (> 5mg/kg) may achieve better therapeutic effect if the appropriate dose is considered in future dose escalation studies. Five previous reports also demonstrated an additive or synergistic effect between TH-302 and therapeutic Abs in a wide range of solid tumors including: glioblastoma 21, 22, 87, osteosarcoma 20, prostate cancer, pancreatic cancer, melanoma, and head and neck cancer 23. In these studies, despite the increase in antitumor activity of therapeutic Ab when combined with TH-302, Ab-related adverse events, such as skin and/or mucosal toxicity, rash, fever, nausea, vomiting, and elevated aminotransferases, were common 20-23, 87. This may explain why naked Ab-based TH-302 combinatorial therapeutics are not sufficient for final approval, and azo-PEGylation-based hypoxia-activated Abs could alleviate systemic toxicity while maintaining high therapeutic efficiency.

Two limitations still exist in the current study. First, immunocompromised xenograft models were used to evaluate Ab-targeting toxicity. This may cause the antitumor efficacy of conditional HAP103 Abs not be precisely estimated. Future work should use immunocompetent hosts to evaluate the preclinical benefit of HAP Abs. Second, for specific targeting hypoxic HCC, comparing the curative effect of HAP103 Ab with that of other advanced therapeutic options (ADCs, CAR-T cells) may be considered, although these agents may not work under hypoxic tumor microenvironment.

In summary, we created a conditional anti-PKM2 Ab for HCC imaging and combination therapy. This hypoxia-activated HAP103 Ab exhibited high targeting selectivity, diminished off-tumor toxicity and more potent anticancer efficacy when combined with the hypoxia-activated prodrug TH-302. This strongly highlights that synergistic hypoxia targeting represents a novel approach to precisely eradicate HCC cells.

## Supplementary Material

Supplementary figures and tables.
