# Hypoxia-activated selectivity-improved anti-PKM2 antibody combined with prodrug TH-302 for potentiated targeting therapy in hepatocellular carcinoma: Erratum

**DOI:** 10.7150/ijbs.128368

**Published:** 2025-12-15

**Authors:** Bo Wang, Fang-Zheng Qi, Ping Chen, Luo-Meng Qian, Hui-Shan Su, Yang Wang, Chen-Hui Wang, Ya-Xin Hou, Qing Zhang, Ding Li, Zhe-Sheng Chen, Si-He Zhang

**Affiliations:** 1 Department of Cell Biology, School of Medicine, Nankai University, Tianjin, 300071, China.; 2 National Clinical Research Center for Cancer, Key Laboratory of Cancer Prevention and Therapy, Tianjin Medical University Cancer Institute and Hospital, Tianjin, 300060, China.; 3 Department of Pharmaceutical Sciences, College of Pharmacy and Health Sciences, St. John's University, Queens, New York, NY, 11439, USA.

In the process of checking the raw data, the authors noticed several inadvertent mistakes occurring in Figure 6E and Figure 7I&7J. During the preparation of these panels, the representative images showing results of H&E staining assays (Figure 6E and Figure 7I), and TUNEL staining assays (Figure 7J) were pasted and placed by mistake. The correct result should be as shown below. The authors apologize for these oversights and declare that this correction does not affect the description, interpretation, or conclusions detailed in the original paper.

## Figures and Tables

**FIGURE 6:**
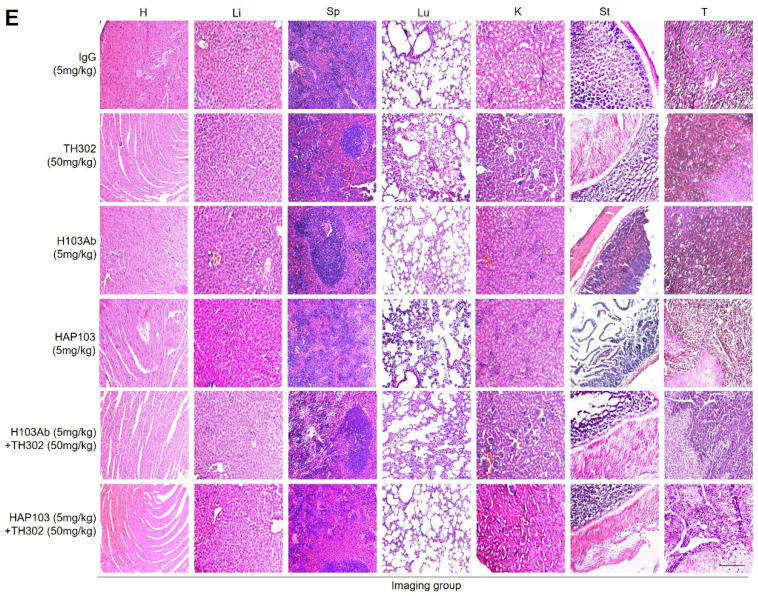
***Ex vivo* identification of the targeting-associated toxicity of HAP103 Ab.** (**E**) Representative H&E staining of main organs and liver cancer tissues from mice subjected to different treatments.

**FIGURE 7:**
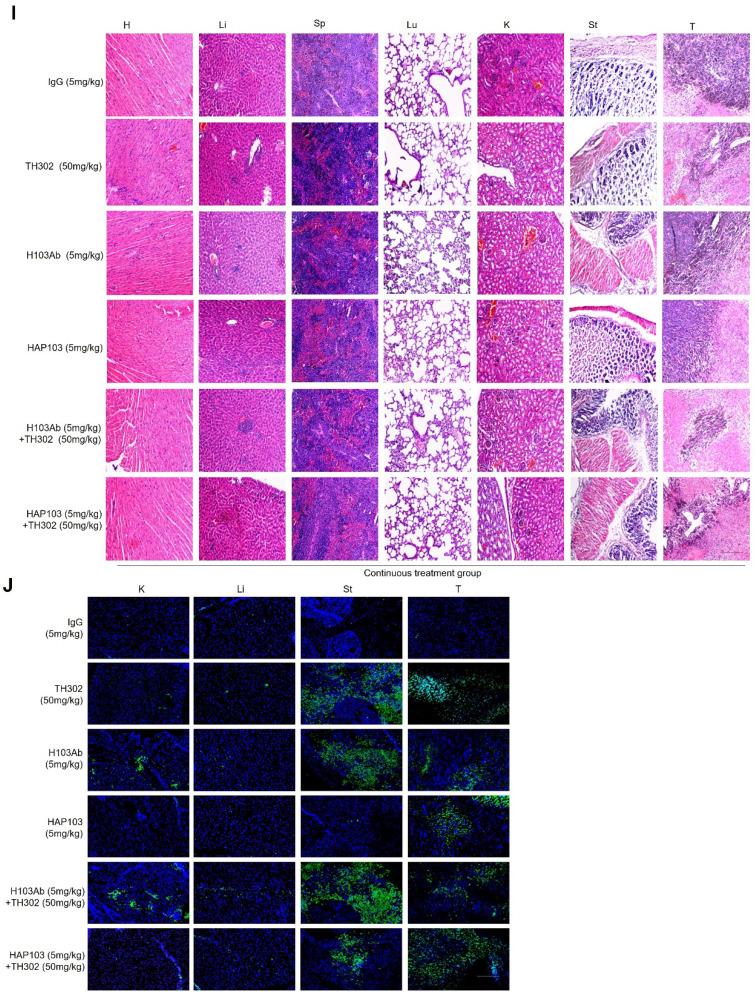
Hypoxia-targeting therapy for liver cancer via the combination of selectivity-improved HAP103 Ab and the prodrug TH-302. (I) Representative H&E staining of main organ tissues from mice subjected to different treatments. (J) Representative TUNEL staining demonstrating the degree of apoptosis in main organs and xenografted HCC tissues after different treatments. Scale bar=500 µm.

