# Supplementary material for: Hypoxia-activated selectivity-improved anti-PKM2 antibody combined with prodrug TH-302 for potentiated targeting therapy in hepatocellular carcinoma
Source: Int J Biol Sci. 2024 Feb 11;20(5):1634–51. doi: 10.7150/ijbs.92211 (PMC10929192; doi:10.7150/ijbs.92211)
Supplement: Supplementary file 1 — Supplementary figures and tables. [file ijbsv20p1634s1.pdf]

Supplementary

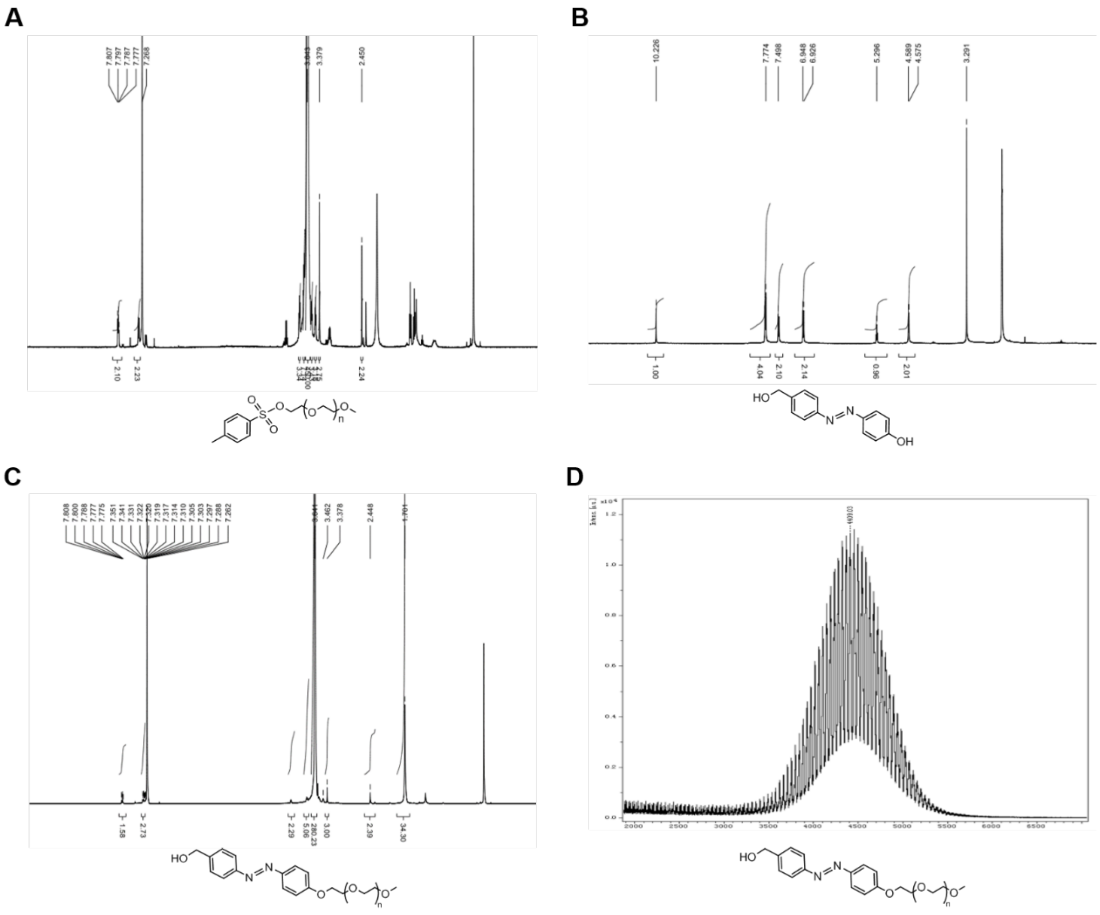

Fig. S1 Identification of intermediate products from PEG5k-azo ester synthesis. The synthesis route of amine-reactive PEG5k-azo ester is illustrated in Fig. 2A. (**A**) ^{1}H NMR spectrum of compound #2. THF-dissolved MPEG5K was added to NaOH. THF-dissolved p-toluenesulfonyl chloride was added, mixed, and stirred for reaction as indicated by thin layer chromatography. After removing insoluble matter, the filtrate was concentrated and the obtained residue was purified by silica gel column chromatography to give compound #2. (**B**) ^{1}H NMR spectrum of compound #5. 4-Aminobenzyl alcohol was added to concentrated HCl. NaNO2 was added to the mixture, followed by the addition of phenol, and the resulting mixture was stirred for 3 h. The pH of the mixture was adjusted to 7.2, and the resulting solid was filtered, washed and dried to give compound #5. (**C**) ^{1}H NMR spectra (δ, ppm, CDCl3, 400 MHz) of compound #6. DMF-dissolved compound #5 and K2CO3 were added to compound 2. The mixture was stirred for 12 h. After removing the solvent, the crude product was purified by silica gel column chromatography to give compound #6. (**D**) The Mass spectrum (MS) analysis of compound #6. Compound #6 was dissolved in dry toluene, after which the solvent was removed. The DCM-dissolved compound was added to the DSC mixture, followed by the addition of triethylamine. The mixture was stirred overnight, and the crude product was purified with a preequilibrated PD10 column to afford compound #7 (PEG5K-azo ester).

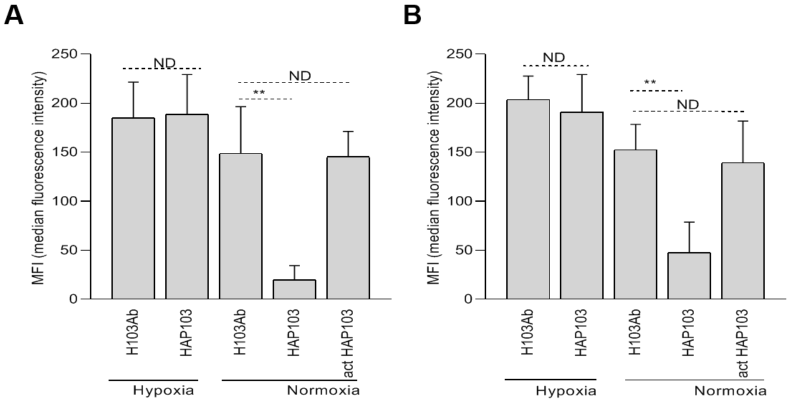

Fig. S2 Flow-cytometry analysis of Abs bound to HepG2 (A) and Huh7 (B) cells cultured under hypoxia and normoxia. n=3. Mean±SD, $^{**}P < 0.01$ vs. H03 Ab. ND: no significant difference. Two-way ANOVA.

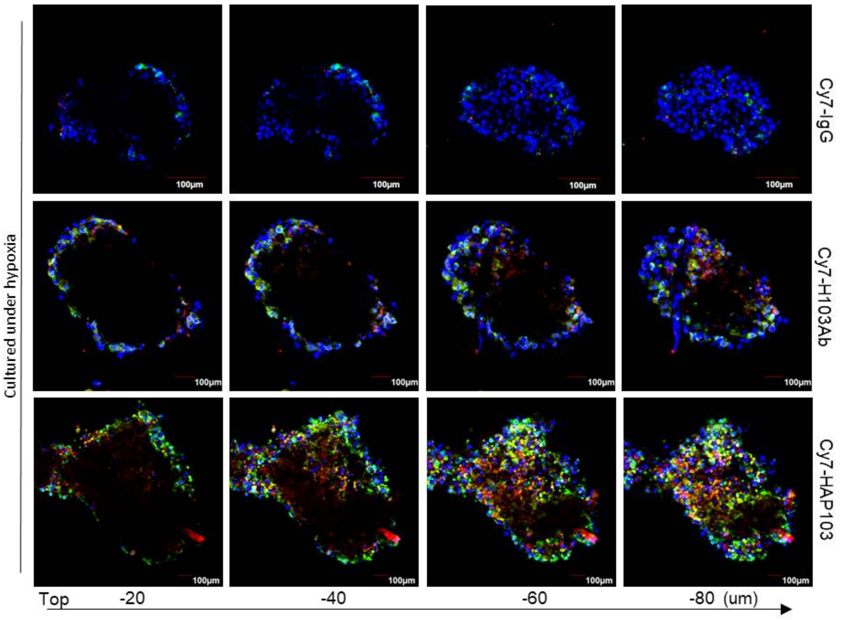

Fig. S3 Confocal imaging of Abs bound to and penetrated hypoxia-cultured Huh7 MCTSs. The fluorescence signals from Cy7-labeled Abs and AF488-directed anti-Hif1a Abs were collected at different levels from the top to the middle of the spheroids on the z-axis.

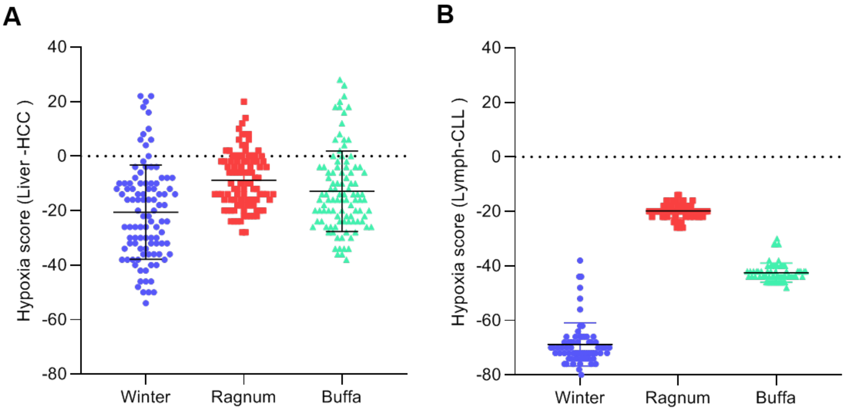

Fig. S4 Hypoxia scores for liver hepatocellular carcinoma (HCC) and chronic lymphocytic leukemia (CLL) according to bioinformatics analysis. Hypoxia scores were calculated for 100 tumor tissues from HCC (**A**) and CLL (**B**) cancer patients. Tumor hypoxia levels were estimated by mRNA-abundance-based signatures developed by Winter et al., Buffa et al. and Ragnum et al (*Nat Commun. 2020,11(1):737*). Briefly, patients with the top 50% of mRNA abundance values for each hypoxia-associated gene in a signature were given a score of +1. Patients with a minimum of 50% mRNA abundance values for that gene were given a score of −1. This process was repeated for every gene in the signature to generate a hypoxia score for each patient, and this process was repeated for each of the three signatures used in this study. High scores suggest that the tumor was hypoxic, and low scores are indicative of normoxia.

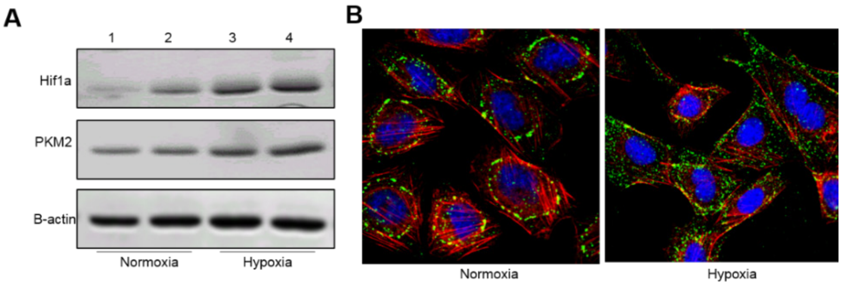

Fig. S5 PKM2 expression under hypoxia and normoxia conditions. (**A**) Western blot analysis of the expression levels of PKM2 and Hif1a in the lysates of Huh7 (1, 3) and HepG2 (2, 4) cells. (**B**) Confocal imaging of the cellular binding of anti-PKM2 Ab. Huh7 cells were fixed, permeabilized, and incubated with mouse anti-PKM2 Ab (1:500) overnight at 4°C, stained with AF488-labeled anti-mouse 2nd Ab (1:1000), and subsequently transferred for visualization via confocal microscopy.

Table S1 Sequence information for H103-VH and -VL for Ab expression.

Deduced VL of H103 Ab from pCANTAB/H103-scFv plasmid:<br>NFMLTQPHSVSESPGKTVTISCTGSSGSIASNYVQWYQQRPGSAPTTVIYEDNQRPPGVPDRFSGSIDSSSN<br>SASLTISALETEDEADYYCQSYDSRNIDVVFGGGTKVTVL
Deduced L-chain of H103 Ab from pcMV3-H103-L plasmid:<br>MGWSCIILFLVATATGVHSNFMLTQPHSVSESPGKTVTISCTGSSGSIASNYVQWYQQRPGSAPTTVIYEDN<br>QRPPGVPDRFSGSIDSSSNSASLTISALETEDEADYYCQSYDSRNIDVVFGGGTKVTVLGQPKAAPSVTLFP

PSSEELQANKATLVCLISDFYPGAVTVAWKADSSPVKAGVETTTPSKQSNNKYAASSYLSLTPEQWKSHRS YSCQVTHEGSTVEKTVAPTECS
Deduced VH of H103 Ab from pCANTAB/H103-scFv plasmid: PGAAAESGPGLVRPSGTLSLICAVSGDSISSSIWWSWVRQSPGKGLEWIGYIYHNGNTYYNPSLESRVTISV DTSENQFSLKLSSVTAADTAVYYCARGYDSSGYYWTDDRYYFDYWGQGTLVTVSS
Deduced H-chain of H103 Ab from pcMV3-H103-H plasmid: MGWSLILLFLVAVATRVLSQVQLQESGPGLVRPSGTLSLICAVSGDSISSSIWWSWVRQSPGKGLEWIGYIY HNGNTYYNPSLESRVTISVDTSENQFSLKLSSVTAADTAVYYCARGYDSSGYYWTDDRYYFDYWGQGTL VTVSSASTKGPSVFPLAPSSKSTSGGTAALGCLVKDYFPEPVTVSWNSGALTSGVHTFPAVLQSSGLYSLSS VVTVPSSSLGTQTYICNVNHKPSNTKVDKKVEPKSCDKTHTCPPCPAPELLGGPSVFLFPPKPKDTLMISRT PEVTCVVVDVSHEDPEVKFNWYVDGVEVHNAKTKPREEQYNSTYRVVSVLTVLHQDWLNGKEYKCKV SNKALPAPIEKTISKAKGQPREPQVYTLPPSRDELTKNQVSLTCLVKGFYPSDIAVEWESNGQPENNYKTTP PVLDSDGSFFLYSKLTVDKSRWQQGNVFSCSVMHEALHNHYTQKSLSLSPGK

Purple characters: variable region; blue characters: constant region; red characters: signal peptide. Green characters: optimized amino acids

Table S2 Exposure pattern of lysine residues in the VH and VL of H103 Ab.

Residues	Solvent-accessible surface area	Connolly surface area	Surface-accessibility (%)
VH			
LYS50	40.5932	177.0814	22.92%
LYS88	29.0309	171.9895	16.88%
VL			
LYS16	67.8505	171.7177	39.51%
LYS108	2.1854	170.2702	1.28%
